# Intraspinal extradural gout tophus in the lumbar vertebral canal: Case reports

**DOI:** 10.1097/MD.0000000000028418

**Published:** 2022-01-07

**Authors:** Zhiqiang Wu, Chunhua Liu, Kehui Dai, Chunfeng Zheng

**Affiliations:** Department of Spinal Surgery, Quanzhou Orthopedic-Traumatological Hospital, Fujian University of Traditional Chinese Medicine, Quanzhou, Fujian Province, China.

**Keywords:** gout tophus, lumbar, vertebral canal

## Abstract

**Rationale:**

Intraspinal gout tophus in the lumbar vertebral canal associated with gouty arthritis is rare. We present 2 cases with the first manifestations of a sequestrated intervertebral disc and an extradural tumor, and histopathologically proven to be gouty deposits in the lumbar vertebral canal.

**Patient concerns:**

The 2 patients presented with typical radiculopathy symptoms and a positive straight leg raise. In 1 case, there was weakness of the left toe extensors, with a positive left femoral nerve traction test. Additionally, the left patellar tendon reflex was weak. In the other patient who was unable to walk, there was a sensory deficit in the saddle distribution.

**Diagnosis:**

Histopathological examination of the specimens taken from the operation confirmed the presence of gouty deposits.

**Interventions:**

Posterior decompression was performed in these 2 cases, and chalky-white materials were identified in the lumbar vertebral canal.

**Outcomes:**

No evidence of neoplasm, infection, or synovial cyst was found.

**Lessons:**

Definitive diagnosis of intraspinal extradural gout tophus, mimicking a sequestrated intervertebral disc or an extradural tumor, may be difficult. The initial suspicion of intraspinal gouty deposits, based on the diagnostic/management algorithm, may effectively avoid incorrect diagnosis via a less invasive procedure than explorative laminectomy.

## 1 Introduction

Gout, resulting from monosodium urate crystal sedimentation, is a common metabolic disorder.^[1,2]^ A large and localized accumulation of urate, commonly developing in individuals with chronic gout, is termed tophi.^[3,4]^ Typically, untreated disease with the formation of tophi progresses to the potential destruction of the peripheral joints. Rarely, the gouty deposits are involved in the spinal canal, and even considerably fewer cases are involved in the lumbar vertebral canal. We present 2 such cases and review the literature on intraspinal extradural gout tophus in the lumbar vertebral canal.

## 2 Case reports

The present study was approved by the Ethics Committee of Quanzhou Orthopedic-Traumatological Hospital. Informed consent was obtained from the patients.

### 2.1 Case 1

A 48-year-old man with alcoholic liver disease, fatty liver disease, and multiple stones in both kidneys presented with sharp back pain for approximately 2 months, which was associated with radiative pain in the bilateral lower extremities. The left leg was worse than the right 1. He had a 10-year history of chronic gouty arthritis and underwent the latest attack approximately 2 weeks before hospital admission. His medications at admission were buxostat (Hengrui Pharmaceutical Co., Lianyungang, China), meloxicam (Dahongying Pharmaceutical Corp., Nibo, China), and pantoprazole sodium (Jiudian Pharmaceutical Co., Changsha, China).

Physical examination revealed that the lumbar lordosis disappeared because of muscle spasms. There was palpable tenderness at the L2–L3 and L4–L5 levels. The supine straight leg raise was 40 bilaterally, and the left femoral nerve traction test was positive. He had grade IV left toe extensor power. Left patellar tendon reflexes were weak. Distal paresthesia or numbness was not observed. A joint examination revealed the presence of small Bouchard's and Heberden's nodes in his hands. Multiple tophus ranging in size from peanut to pigeon egg can be seen in both hands, feet, knees, and ankles.

**Figure 1:**
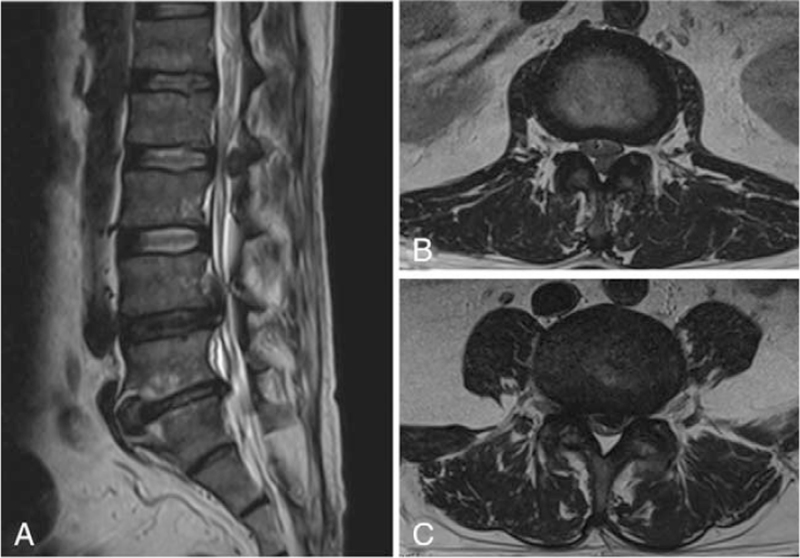
Figure 1–2 Images illustrate the patient of a 48-year-old man with gouty deposits. (1) Preoperative sagittal (A) and coronal (B) MR images showing a 2-cm left-sided extradural lesion at the L2-L3 intervertebral level. Preoperative sagittal and coronal MRI images (A, C) showing a mass at the L4 vertebral level. (2) The histopathological examination confirmed the deposition of amorphous material, surrounded by macrophages and fibroblasts.

Routine radiography of the lumbar spine was normal. Magnetic resonance imaging (MRI) showed a 2-cm left-sided extradural lesion in the posterior epidural space (Fig. 1A and 1B) that was located at the L2-L3 intervertebral level, and a mass at the L4 vertebral level on the left side, extending toward the midline to the right (Fig. 1A and 1C). Both masses caused moderate lumbar spinal stenosis. Laboratory examination included white blood cell count, 9.65 10^^9^/L; erythrocyte sedimentation rate, 94 mm/h; C-reactive protein 89 mg/L; blood urea nitrogen, 3.6 mmol/L; creatinine, 76.1 umol/L; serum uric acid, 651.2 umol/L. These findings were interpreted as being consistent with radiological diagnoses of a sequestrated intervertebral disc at the level of L4 and an extradural tumor at the L2-L3 level.

L2 laminectomy was performed. A chalky-white mass compressing the L3 nerve root and theca was observed in the posterior epidural space, which pushed the dura from the left side to the right. Subsequently, the mass was then removed. Similarly, L4 laminectomy was performed. A sequestrated disc compressing the L5 nerve root and theca was identified and located in the posterior epidural space. The fragments were then removed.

**Figure 2:**
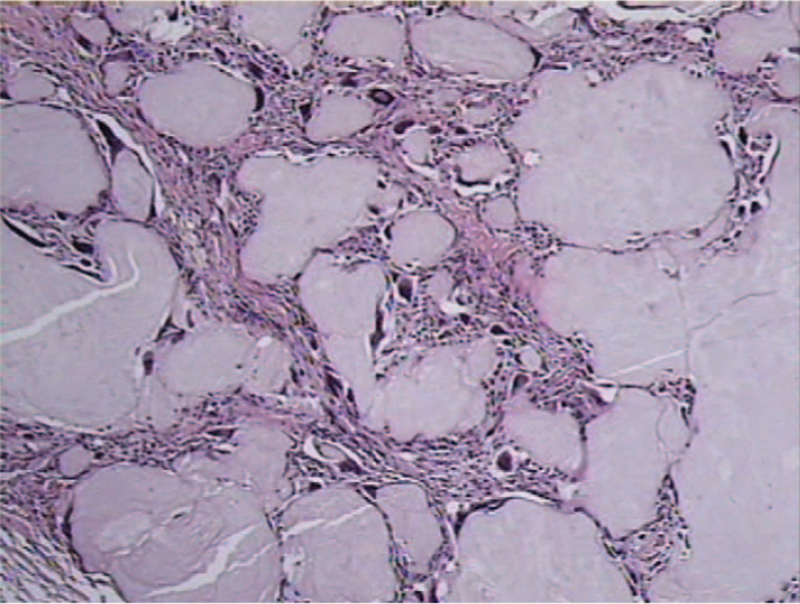
The histopathological examination confirmed the deposition of amorphous material, surrounded by macrophages and fibroblasts.

The histopathology of the specimens, extracted from the L2/3 intervertebral level, confirmed the chalky-white lesion containing deposits of tophaceous gouty material, as shown in Figure 2. However, histopathological examination confirmed that the epidural mass at the L4 vertebral level was the sequestrated intervertebral disc. The patient's intractable back and leg pain was completely relieved when he was followed up in the outpatient clinic.

### 2.2 Case 2

A 63-year-old man with grade 3 hypertension, atherosclerotic artery disease, fatty liver disease, and arrhythmia experienced frequent repeated back pain for approximately 2 years and increasingly radiating leg pain. He complained of numbness in the saddle area. These symptoms caused him to be unable to walk normally. He also had a 30-year history of gouty arthritis, with the most recent onset symptoms dating to 6 months prior to admission. Currently, the patient's medications are the use of analgesics and stomach-protecting agents, with a specific focus on which drug is unclear.

On examination, he had mild tenderness on palpation of the L4–L5 level. The supine straight leg raise was limited to 30 on the left side. There was a sensory deficit in the saddle distribution, with essential normal deep tendon reflexes in the lower extremities. Multiple scattered circular subcutaneous nodules were seen in the extremities, with a diameter of approximate 0.5 to 2.0 cm. No local tenderness, redness, or swelling symptoms were observed, and skin temperature was normal.

**Figure 3:**
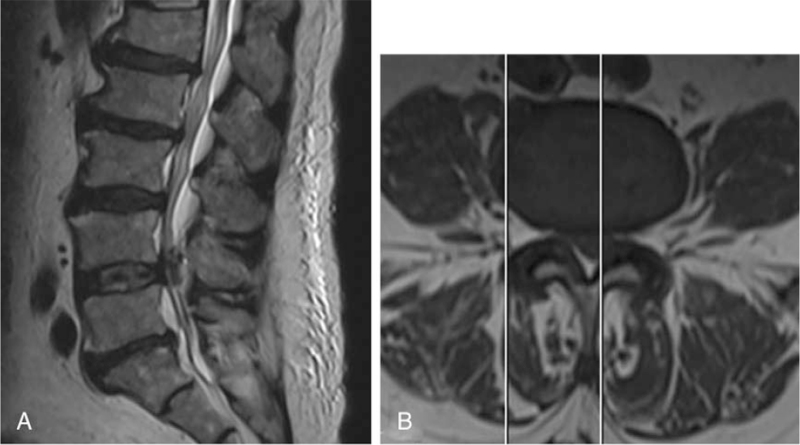
Fig. 3–6 Images illustrate the patient of a 63-year-old man with gouty deposits. (3) Preoperative sagittal (A) and coronal (B) MR images and CT (4) showing posterior root and theca compression at the L4-L5 intervertebral level. (5) The histopathological image showed tophaceous gout surrounded by granuloma formation with macrophages and fibroblasts. (6), showing no recurrence of intraspinal gouty deposits during follow-up.

**Figure 4:**
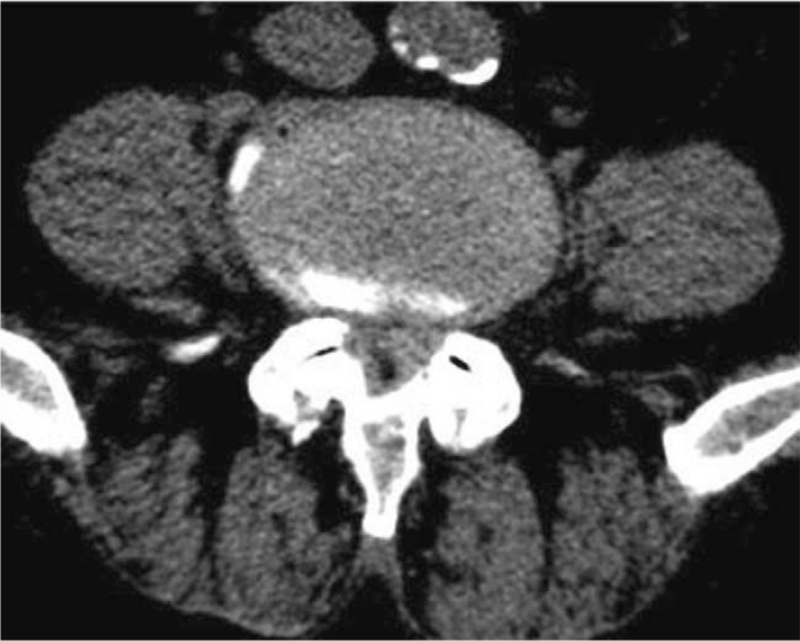
CT showing a posterior root and theca compression at L4-L5 intervertebral level.

Routine radiography revealed lumbar vertebral degeneration without erosive changes. MRI and computed tomography (CT) revealed a posterior root and theca compression at the L4–L5 intervertebral level on the left side (Figs. 3 and 4), which resulted in significant lumbar spinal stenosis with neurological syndromes. Laboratory examination included white blood cell count, 3.38 10^^9^/L; erythrocyte sedimentation rate, 15 mm/h; C-reactive protein 16.3 mg/L; blood urea nitrogen, 5.6 mmol/L; creatinine, 129.0 umol/L; serum uric acid, 674.0 umol/L. A radiographic diagnosis of “sequestrated lumbar intervertebral disc” was made.

**Figure 5:**
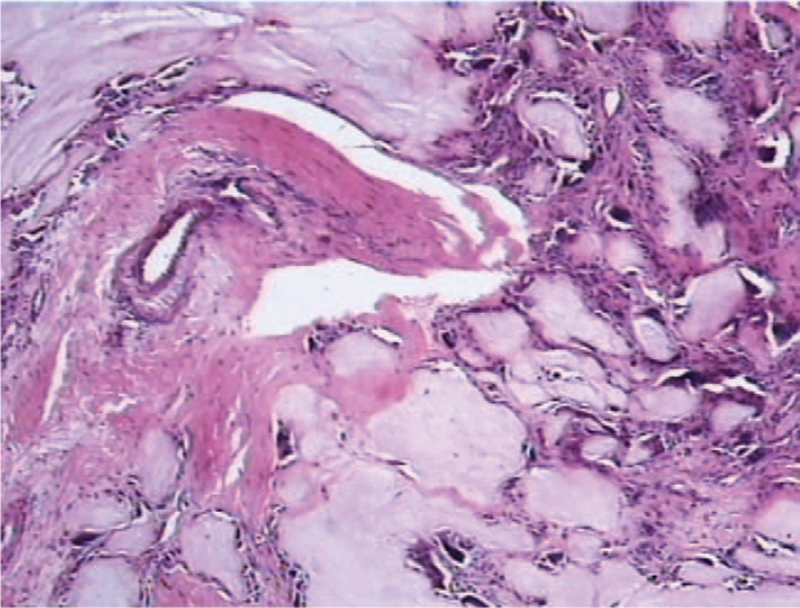
The histopathological image showed the tophaceous gout surrounded by granuloma formation with macrophages and fibroblasts.

**Figure 6:**
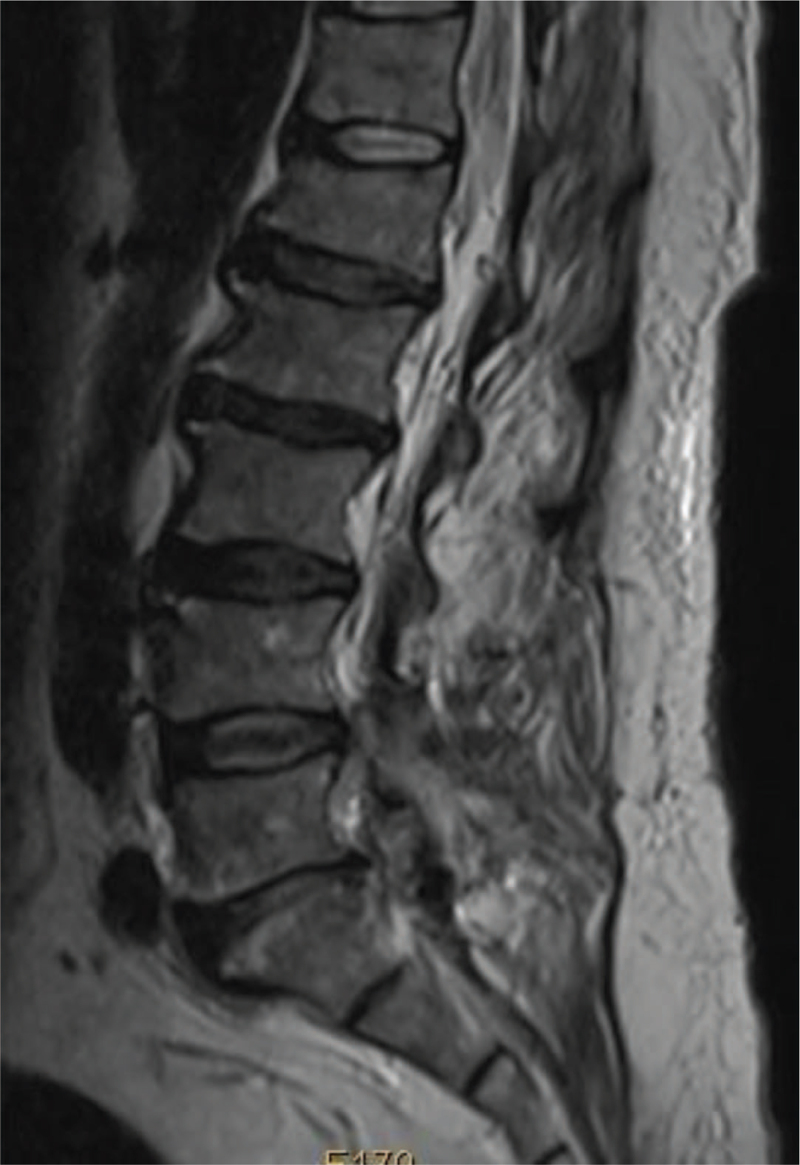
Showing no recurrence of intraspinal gouty deposits during the follow-up.

Posterior decompression was performed at L4–L5. The chalky-white materials were identified and found to erode the left L4–L5 facet joint. The left theca was pushed to the right side. Subsequently, the fragment was removed, and the compression was completely relieved. A histopathological examination confirmed the chalky-white materials to be the urate crystals sedimentation, as shown in Figure 5. After the operation, follow-up examinations performed at 3 and 6 months exhibited no additional neurological symptoms, without the recurrence of intraspinal gouty deposits, as shown in Figure 6.

## 3 Discussion

Intraspinal extradural gout tophus occurring in the lumbar spine can be present in a variety of ways. Typical clinical symptoms, such as medically intractable low back pain and/or neurological deficit, are not specific to tophaceous deposits and can develop in a short period of time or progress slowly over many years.^[5,6]^ Therefore, we believe that the major concern for neurosurgeons is the proposal of an appropriate diagnostic/therapeutic strategy when faced with these cases.

Usually, this pattern of intraspinal gout tophus is confused with other spinal canal space-occupying lesions, such as abscess, disc migration, neoplasms, hematoma, and synovial cysts.^[3,7,8]^ In the initial care plan, routine radiography is appropriate and cost-effective. Although this preliminary screening tool lacks sensitivity,^[9]^ it can identify lumbar instability associated with calcified gout. When individuals present with medically refractory axial-associated pain or neurological compromise, more specific imaging examinations, such as CT and MRI, are generally required. Importantly, MRI with gadolinium protocol becomes increasingly necessary not only to aid us in diagnosing the posterior gouty tophi, but also to avoid misdiagnosis of herniated discs, particularly pathological lesions, for example, neoplasms, infections, and synovial cysts.

In-depth assessment of any patients with lumbar intraspinal gout tophus should contain a detailed disease history and comprehensive physical examination, since these individuals most commonly have an extensive history of alcoholism, hyperuricemia, chronic gout, or renal insufficiency.^[5,10]^ Among them, the white blood cell count, erythrocyte sedimentation rate, C-reactive protein, and serum uric acid levels are usually elevated,^[3,5,10]^ which may further provide the diagnostic possibilities of gout tophus previously not considered. Unfortunately, these characteristics could not exclude infections from differential diagnosis. Thus, vigilance is critical, especially when the patient develops a fever.

In individuals who do not have progressive nerve/spinal cord compression, non-operative intervention may be a reasonable therapeutic alternative, especially when surgical risk is high. A needle biopsy of the abnormal bone or disc space can reliably establish the presence of urate crystal sedimentation and avoid any further invasive surgical intervention.^[11,12]^ However, patients with medically refractory axial pain, or those who present with remarkable neurological deterioration and imageology evidence should be considered when performing surgical decompression in a timely manner. In addition, surgical stabilization is recommended if necessary. Our 2 patients, for example, had the aforementioned symptoms. Given the serious impact on the patient's daily life, we selected a surgical intervention. Fortunately, in both of them, satisfactory outcomes were obtained after operation.

The typical imaging findings of spinal gout are generally lobular-shaped calcified masses with increased density, and are potentially accompanied by well-defined erosive bone destruction with sclerotic borders.^[11,13]^ In our 2 cases, it was not easy to clarify the diagnosis of intraspinal tophaceous deposits on CT or MRI, since the osseous erosions of the vertebra and the increased density of the gout tophus were not evident. Therefore, identification of intraspinal involvement in these 2 cases, following an appropriate diagnostic/management algorithm, was conducive for neurosurgeons to make an exact presumptive diagnosis.

## Author contributions

**Conceptualization:** Zhiqiang Wu, Chunhua Liu.

**Investigation:** Zhiqiang Wu, Chunhua Liu, Kehui Dai, Chunfeng Zheng.

**Methodology:** Zhiqiang Wu, Chunhua Liu, Kehui Dai.

**Project administration:** Zhiqiang Wu.

**Resources:** Chunhua Liu.

**Supervision:** Zhiqiang Wu, Chunhua Liu.

**Writing – original draft:** Zhiqiang Wu, Kehui Dai, Chunfeng Zheng.

**Writing – review & editing:** Zhiqiang Wu, Chunhua Liu.
